# Advancing the prediction of factors associated with bipolar disorder risk: utilizing early recognition tools and polygenic risk scores

**DOI:** 10.1186/s40345-025-00404-8

**Published:** 2025-12-10

**Authors:** Silvia Biere, Silke Matura, Kristiyana Petrova, Fabian Streit, Andreas G. Chiocchetti, Kira F. Ahrens, Charlotte Schenk, Michael M. Plichta, Raffael Kalisch, Michèle Wessa, Viola Oertel, Andrea Pfennig, Michael Bauer, Philipp Ritter, Thomas G. Schulze, Christoph U. Correll, Andreas Bechdolf, Klaus Lieb, Oliver Tüscher, Sarah Kittel-Schneider, Andreas Reif, Thorsten M. Kranz

**Affiliations:** 1 Department of Psychiatry, Psychosomatic Medicine and Psychotherapy, University Hospital, Goethe University, Heinrich-Hoffmann-Str. 10, 60528 Frankfurt am Main, Germany; 2 Department of Psychiatry and Psychotherapy, Medical Faculty Mannheim, Central Institute of Mental Health, Heidelberg University, Mannheim, Germany; 3 Hector Institute for Artificial Intelligence in Psychiatry, Medical Faculty Mannheim, Central Institute of Mental Health, Heidelberg University, Mannheim, Germany; 4 German Center for Mental Health (DZPG), Partner Site Mannheim-Heidelberg-Ulm, Mannheim, Germany; 5 Department of Child and Adolescent Psychiatry, Psychosomatics and Psychotherapy, Autism Research and Intervention Center of Excellence Frankfurt, University Hospital Frankfurt, Goethe University, Frankfurt am Main, Germany; 6 Institute of Psychology, Leibniz Institute for Resilience Research (LIR), Technical University of Darmstadt, Mainz, Darmstadt, Germany; 7 Neuroimaging Center (NIC), Focus Program Translational Neuroscience (FTN), Johannes Gutenberg University Medical Center Mainz, Mainz, Germany; 8 Department of Neuropsychology and Psychological Resilience Research, Medical Faculty Mannheim, Central Institute of Mental Health, Heidelberg University, Mannheim, Germany; 9 DKFZ Hector Cancer Institute at the University Medical Center, Mannheim, Germany; 10 Division Cancer Survivorship and Psychological Resilience, German Cancer Research Center (DKFZ), Heidelberg, Germany; 11 Department of Psychiatry and Psychotherapy, Faculty of Medicine, Carl Gustav Carus University Hospital, Dresden University of Technology, Dresden, Germany; 12 Institute of Psychiatric Phenomics and Genomics, University Hospital Munich, Ludwig- Maximilians-University of Munich, Munich, Germany; 13 Department of Child- and Adolescent Psychiatry, Charité Universitätsmedizin Berlin, Berlin, Germany; 14 Department of Psychiatry, The Zucker Hillside Hospital, Northwell Health, Glen Oaks, NY USA; 15 Department of Psychiatry and Molecular Medicine, Donald and Barbara Zucker School of Medicine at Hofstra/Northwell, Hempstead, NY USA; 16 Department of Psychiatry, Psychotherapy and Psychosomatics, Vivantes Hospital at Urban and Vivantes Hospital at Friedrichshain, Berlin, Germany; 17 Department of Psychiatry and Psychotherapy, Charité Universitätsmedizin Berlin, Campus Charité Mitte, Berlin, Germany; 18 Department of Psychiatry and Psychotherapy, Johannes Gutenberg University Medical Center, Mainz, Germany; 19 Leibniz Institute for Resilience Research, Mainz, Germany; 20 Institute of Molecular Biology (IMB) Mainz, Mainz, Germany; 21 Department of Psychiatry and Neurobehavioural Science, University College , Cork, Ireland; 22 Department of Psychiatry, Psychosomatic Medicine and Psychotherapy, University Hospital Wuerzburg, Wuerzburg, Germany; 23 Fraunhofer Institute for Translational Medicine and Pharmacology ITMP, Theodor- Stern-Kai 7, 60596 Frankfurt am Main, Germany

**Keywords:** Polygenic risk score, Bipolar disorder, Early recognition, Early symptoms, Risk factors, Family history

## Abstract

Bipolar disorder (BD) is a highly heritable mental illness that affects ∼ 1–2% of the world’s population and has complex genetic and environmental underpinnings. Early detection is critical to improving treatment outcomes, but current strategies have limited predictive power. Early detection tools such as the Early Phase Inventory for Bipolar Disorder (EPI*bipolar*) and the Bipolar At-Risk (BARS) criteria assess phenotypic risk factors, including family history (FH) and subthreshold mood problems. Polygenic risk scores (PRS) are a quantitative metric of genetic susceptibility. This study examined the associations between BD-PRS and screening tools in order to assess their combined potential to identify individuals at risk of BD with improved predictive accuracy. The analysis included 1068 participants, including 199 at-risk young adults aged 15 to 35 years and 869 healthy controls aged 18 to 50 years. All of them had no prior psychiatric disorders. Inclusion criteria for the at-risk group comprised a positive FH (1st or 2nd degree) for BD, major depressive disorder (MDD), attention-deficit/hyperactivity disorder (ADHD), or the presence of specific BD risk factors (e.g., subthreshold hypomanic symptoms, mood swings, or sleep disturbances). Participants who had a confirmed BD, schizophrenia, schizoaffective disorder diagnosis, or other psychiatric conditions that could explain the symptomatology, were excluded. Diagnostic assessments that were utilized validated early detection instruments, including EPI*bipolar*, Bipolar Prodrome Interview and Symptom Scale-Prospective (BPSS-FP), and BARS criteria. Binary logistic regression models were employed to assess associations between BD-PRS and phenotypic risk markers, with adjustments for population stratification. Results revealed significant associations between BD-PRS and BARS criteria risk groups and EPI*bipolar* “at risk” criteria compared to controls. Significant associations were also identified for subscales including FH for BD, MDD, or schizophrenia, sleep and circadian rhythm disturbances, depressive characteristics, functional impairment, and episodic course. However, no significant associations were observed between BD-PRS and BPSS-FP, which highlights variability in the sensitivity of different early detection instruments. Our findings emphasize the potential of combining genetic susceptibility measures with phenotypic risk markers to enhance early detection strategies for BD. Further research is needed to optimize predictive models and evaluate the clinical utility of PRS in early intervention frameworks.

**Supplementary Information:**

The online version contains supplementary material available at 10.1186/s40345-025-00404-8.

## Introduction

Bipolar disorder (BD) is a highly heritable mental health illness with a multifactorial etiology including genetic and environmental influences. The disorder often manifests during adolescence or early adulthood and is characterized by a chronic course with recurrent mood episodes (Cirone et al. 2021). BD affects approximately 1–2% of the global population (Rowland and Marwaha 2018). The World Health Organization (2024) recognizes BD as one of the leading causes of disability worldwide with significant personal and socioeconomic impacts (Mullins et al. 2021).

Early detection of BD is critical for timely intervention. It can reduce mood episode severity, prevent misdiagnosis, and improve long-term outcomes (Vieta et al. 2018). Early intervention delays the onset of full episodes. Furthermore, it enhances treatment efficacy, while also reducing suicidality, improving treatment adherence, and enhancing quality of life (Kalman et al. 2018; Pfennig et al. 2020). On average we see a delay of six years between BD onset and treatment initiation (Lublóy et al. 2020), even though observations from early detection centers indicate that individuals at high risk often present with subsyndromal symptoms long before full manifestation (Dagani et al. 2017; Leopold et al. 2014; Pfennig et al. 2020). These precursor symptoms — such as mood instability, subthreshold manic or depressive episodes, sleep disturbances, anxiety, and ADHD as a risk factor — are strong predictors of BD development. This underscores their importance in early diagnosis (Biere et al. 2020; Faedda et al. 2019; Prunas et al. 2019; Van Meter et al. 2016). In response to this, researchers have developed early detection instruments such as EPI*bipolar* (Leopold et al. 2012; Pfennig and Leopold 2010), BPSS-FP (Correll et al. 2014), and BARS criteria (Bechdolf et al. 2012; Fusar-Poli et al. 2018). Those instruments have shown promising results in identifying high-risk individuals and enabling the detection of subsyndromal symptoms prior to the full manifestation of BD (Bechdolf et al. 2014; Correll et al. 2014; Fusar-Poli et al. 2018; Lee et al. 2023). Additionally, to the clinical phenotype, EPI*Bipolar* also includes family history (FH) as a central predictor, due to its relevance in early risk assessment (Pfennig et al. 2020). FH is the strongest known predictor of BD risk and encompasses both genetic and non-genetic risk factors (Craddock and Sklar 2013; Scott et al. 2017). Due to BD’s heritability rate of up to 70% (Stahl & Bipolar Working Group of the Psychiatric Genomics Consortium, 2019), understanding the genetic underpinnings of BD-specific symptoms may provide valuable insights for refining early detection strategies and for potentially improving diagnostic approaches.

Genome-wide association studies (GWAS) have proven to be a helpful method to further clarify disease etiology. GWAS identify genetic variants, particularly common single-nucleotide polymorphisms (SNPs), across the genome that are associated with disease susceptibility because of a complex mode of inheritance. Polygenic risk scores (PRS) can be computed resulting in an estimate of an individual’s genetic predisposition to the disorder based on GWAS on BD (Mullins et al. 2021; Stahl, 2019). PRS aggregate the effects of BD-associated SNPs and offer a way to quantify genetic burden. However, PRS alone explain only 4% − 5% of the phenotypic variance, and are not sufficient to predict BD on their own (Mullins et al. 2021; O’Connell et al. 2019; O’Connell and Coombes 2021). Despite their limitations PRS could still be a meaningful prediction instrument when it comes to understanding genetic risk once they are combined with other clinical assessment tools. Mars et al. (2022) showed that FH and PRS offer partially independent, non-interchangeable insights into disease risk. This integrated approach — assessing both genetic (PRS) and phenotypic factors (FH and other risk factors) — could improve the early identification of individuals who are at high risk for BD. This approach could potentially also allow for interventions before severe symptoms occur (O’Connell and Coombes 2021). The optimal method for combining these two measures, however, is still unclear. A framework that incorporates both genetic data from PRS and phenotypic insights holds the potential to transform early detection and intervention approaches. Combining those two approaches could lead to more tailored prevention measures, and it could ultimately reduce the disease burden of BD (Mistry et al. 2018).

The present study investigates whether BD-PRS is associated with specific prodromal risk constellations as assessed by validated early detection tools. To clarify the relationship between genetic susceptibility and phenotypic risk markers by focusing on these associations may contribute to a deeper understanding of the correlation between genetic and clinical factors in individuals at risk for BD, and they may provide a basis for refining early detection strategies.

## Materials and methods

### Experimental procedures and study setting

The present study included a total sample of 1068 participants. The study sample of 199 young adults, aged 18 to 35, was recruited for the multicenter prospective, naturalistic BipoLife sub-study entitled “Improving Early Recognition and Intervention in Individuals at Risk for Developing Bipolar Disorder (BD)” (Early-BipoLife). This study longitudinally tracks young, help-seeking individuals over a three-year period (Pfennig et al. 2020; Ritter et al. 2016). Recruitment took place between July 2015 and September 2018 at nine sites across Germany (Berlin - two sites: CCM, Charité Universitätsmedizin Berlin, Vivantes Hospital at Urban; Bochum, Dresden, Frankfurt/Main, Hamburg, Marburg, Brandenburg/Neuruppin, Tübingen). The coordinating center for the study is the Department of Psychiatry and Psychotherapy at the University Hospital of TUD Dresden University of Technology.

Adolescents and young adults who sought help at early intervention centers or initiatives and who had at least one of the identified risk factors for BD, as well as inpatients and outpatients with depressive syndromes or ADHD, were recruited. All diagnostic interviewers participated in a two-day training program that covered the study’s objectives, design, and methodology. They were continuously supervised by experienced supervisors throughout the course of the study to ensure consistency and reliability in data collection. This study was supported by the German Federal Ministry of Education and Research (BMBF, grant number: 01EE1404A). Ethics approval was obtained from the Medical Faculty at Dresden University of Technology (TUD) (No. EK290082014) as well as the respective ethics committees of all participating sites. Written informed consent was obtained from all participants (or legal guardians of underaged participants), and their understanding of the study protocol was confirmed. The study was conducted in accordance with the Declaration of Helsinki (Association 2013) and the standards of good clinical practice (GCP).

The control group consisted of 869 healthy individuals aged 18 to 50 with no prior history of mental disorders and no current mental disorder symptoms. These participants were drawn from the Longitudinal Resilience Assessment (LORA) study serving as a consistent sample for genetic control purposes (Biere et al. 2020; Chmitorz et al. 2021).

### Sampling

Early-BipoLife participants were screened on inclusion and exclusion criteria and categorized into three groups:

(1) Help-seeking individuals positively screened for an increased risk for BD (screenBD at-risk): This group included participants fulfilling one or more early detection risk factors: positive family history (1st or 2nd degree relative with a confirmed diagnosis of BD, MDD, schizoaffective disorder or schizophrenia), subthreshold hypomanic symptoms, mood swings, sleep/circadian disturbances, or subclinical depressive symptoms (for a complete list see Pfennig et al. 2020).

(2) Individuals diagnosed with MDD based on the criteria outlined in the DSM-IV or DSM-5.

(3) Individuals diagnosed with ADHD based on the criteria outlined in the DSM-IV or DSM-5.

Based on these criteria, all included participants were categorized as screened BD at-risk participants. Individuals meeting the diagnostic criteria for BD, such as schizoaffective disorder, or schizophrenia, as well as those with primary substance abuse, anxiety disorders, or obsessive-compulsive disorder, were excluded. A comorbid personality disorder did not constitute an exclusion criterion.

Since BipoLife did not include a control cohort for genomic biomarkers, we utilized the healthy control cohort from the LORA study. This control cohort provided genome-wide genotyping data and comprised individuals aged 18 to 50 years with normal or corrected vision, sufficient German language skills, and the ability to provide informed consent. All healthy controls were first screened for current and past psychiatric disorders using the Mini-International Neuropsychiatric Interview (M.I.N.I.). Individuals were excluded if they had a lifetime diagnosis of schizophrenia or BD, an organic mental disorder, substance dependence (excluding nicotine), a severe current Axis I disorder, a serious medical or neurological condition, or a known learning disability. Recent participation in a drug trial (within the past six months) also led to exclusion. In addition, standardized case report forms were used to record sociodemographic information, general medical history, and family history of psychiatric disorders. Family history data were collected for descriptive purposes but were not used as an exclusion criterion. BD at-risk participants were compared to all control individuals, excluding those who tested negative on the respective scales from the analyses.

### Instruments

Structured diagnostic interviews and standardized tools were used to assess inclusion criteria. Diagnoses of MDD and ADHD were determined using the Structured Clinical Interview for DSM-IV Axis I Disorders (SCID-I; Wittchen et al. 1997) and clinical confirmation, respectively. To evaluate the potential risk factors/constellations for conversion, structured early detection instruments, including EPI*bipolar* (Leopold et al. 2012; Pfennig and Leopold 2010), BPSS-FP (Correll et al. 2014) and BARS criteria (Bechdolf et al. 2012; Fusar-Poli et al. 2018) were applied.

EPI*bipolar* is a diagnostic framework developed to systematically evaluate risk factors for BD, drawing on evidence from clinical studies and practical experience. It examines key risk indicators by incorporating data from patient histories, SCID interviews, and BPSS-FP assessments, including family history of BD, increasing cyclothymia, hypomanic syndrome, specific sleep and circadian rhythm disorders, consistent cyclothymia, depressive characteristics, ADHD, family history of schizophrenia or MDD, current MDD, functional impairments, episodic course, and substance misuse. Based on this comprehensive evaluation, individuals are categorized into risk, high-risk, and ultra-high-risk categories for conversion to BD.

The BPSS-FP is a structured diagnostic tool designed to assess prodromal symptoms and the risk of developing BD. It evaluates subsyndromal symptoms, including mood instability, sleep disturbances, and functional impairments, and identifies two specific syndromes: Attenuated Mania Symptom Syndrome (AMSS), characterized by subthreshold manic symptoms, and Genetic Mania Risk and Deterioration Syndrome (GMRDS), which combines genetic vulnerability with functional decline. The BPSS-FP enables early risk stratification with good internal consistency, convergent validity and inter-rater reliability (Correll et al. 2014).

The BARS criteria, developed by Bechdolf et al. (2012), is a set of ultra-high-risk criteria aimed at identifying individuals aged 15–24 years at high risk for developing BD. These criteria encompass four distinct at-risk groups: subthreshold mania, characterized by hypomanic symptoms that do not meet full diagnostic thresholds; depression with cyclothymic features, involving major depressive episodes accompanied by a cyclothymic temperament; depression with genetic risk, defined as major depressive episodes combined with a FH of BD or related conditions; and mixed symptoms, which was introduced as an extension by Falkenberg’s group, describing the co-occurrence of depressive and hypomanic symptoms.

Comorbid disorders, psychiatric treatment, physical illness, and substance use were assessed using standardized Case Report Forms. The burden of disease and psychosocial functioning were evaluated using the Global Assessment of Functioning Scale (GAF; Hall 1995) and the Functioning Assessment Short Test (FAST; Rosa et al. 2007). For more detailed information on the psychometric properties of the instruments, predictors of BD-related outcomes, and additional factors such as risk and resilience markers, as well as the longitudinal course of BD development, see Martini et al. 2024; Pfennig et al. 2020. In order to rule out any Axis I disorder (based on DSM-IV criteria) current or past psychiatric symptoms in the healthy control group were screened via the Mini-International Neuropsychiatric Interview (M.I.N.I.) (Lecrubier et al. 1997; Sheehan et al. 1992).

### Genotyping, quality control and imputation

Genotyping for *n* = 353 participants was performed using the Global Screening Array SA (GSA, Multi-Disease (MD). Version 3.0) at the Life & Brain GmbH Platform Genomics, Bonn, Germany. The genotyping sample was drawn from participants across the broader project, but this manuscript focuses specifically on individuals from the Early BipoLife substudy. Genotyping of *n* = 958 participants of the LORA study (control cohort) was carried out on a GSA-MD V 1.0 at the Broad Institute in Cambridge, Massachusetts, USA. Quality control of all subjects was performed using PLINK v1.9 (Ahrens et al. 2022; Chang et al. 2015). In both datasets, SNPs were filtered to exclude those with minor allele frequencies ≤ 0.01, calling rate of ≤ 0.98, variants deviating from Hardy–Weinberg-Equilibrium (HWE) (*p* < 1 × 10 − 6), and tri-allelic variants or variants not uniquely mappable. Participants were excluded in case of missingness > 0.02, heterozygosity rate > 0.2, and sex mismatch. Filtering for population structure and relatedness was carried out on a selected high-quality (HWE *p* <.02, MAF > 0.2, missingness = 0) SNP set that was LD pruned (r² = 0.1). In case of cryptically related subjects (pi hat > 0.2), one of the subjects was excluded, preferentially retaining cases. To assess hidden population stratification, principal component analysis (PCA) was performed, and outliers from the HapMaP CEU reference panel were excluded. After quality control, the datasets were merged and another iteration of quality control and PCA was carried out as described above, including correct mapping of the SNPs regarding strand orientation, chromosomal position and unique ID mapping. In total, 154 participants from the Early-BipoLife cohort and 89 persons from the LORA cohort were excluded from the subsequent analyses. Those participants either belonged to different substudys of Bipo-Life, did not pass the criteria for genetic quality control including ethnicity outliers, or displayed missing information on clinical parameters (both cases and controls).

The final dataset was composed of *n* = 199 participants from the Early-BipoLife study (case sample) and *n* = 869 participants from the LORA cohort (control sample). Genetic imputation was performed on the Michigan Imputation Server (Das et al., 2016) using 1000 g phase 3 v5 reference panel, population EUR, INFO > 0.8 and a rsq filter < 0.3 for both independent datasets. After imputation, the two datasets (case, control) were merged using Plink v1.9 (as above) and another iteration of QC was performed. The final dataset was used for polygenic risk score calculation.

### Calculation of polygenic risk scores (PRS)

PRS calculation was performed using PRSice software version 2.3.5 with default options [clump-kb 250, clump-p 1.0, clump r2 0.1, interval 5e-05, lower 5e-08, stat BETA] (Choi and O’Reilly 2019). PRS calculation for BD was based on the summary statistic files of the second Psychiatric Genomics Consortium Bipolar Disorder (PGC-BD) GWAS (Mullins et al. 2021), using an INFO score filtering (INFO > 0.8). There was no overlap between the present study sample and the used BD discovery sample. BD-PRS were z-transformed based on the mean and standard deviation observed in the control group. We applied the best-fit thresholding approach of PRSice. To determine the best fit with the BD phenotype, the largest variance explained was calculated using Nagelkerke’s pseudo-R² of the full model for Early-Bipo-Life vs. LORA. This model included BD-PRS alongside covariates such as the first five principal components for adjustment for population stratification. Its performance was compared to the null model, which incorporated only the covariates (Choi et al. 2018).

### Statistics

All subsequent analyses were conducted using SPSS version 29.0 for Windows (IBM Corp., USA). Binary logistic regression models were employed to assess whether BD-PRS (the independent variable) was linked to phenotypic markers of early bipolar disorder as compared to the control group (the dependent variable). Odds ratios (ORs) corresponding to the increase of one standard deviation (SD) in BD-PRS are presented. The regressions accounted for the first five principal components to adjust for potential population stratification. Unadjusted p-values are shown. For the fourteen separate binary logistic regression models, the effect size was calculated using GPower version 3.1.9.7, incorporating 12 EPIbipolar risk factors, 1 BPSS-FP criterion, and 1 BARS criterion as predictors.

## Results

Results for BD-PRS calculation based on the complete sample (*n* = 199 screen BD at risk and *n* = 869 healthy controls) according to the criteria described in the Material and Methods section were as follows: the best-fit results were observed for a p-value threshold = 0.04, variance explained by the polygenic risk score (PRS.R2) = 0.016, Variance explained by the polygenic risk score adjusted for prevalence of BD in the general population (PRS.R2.adj) = 0.009, variance explained by the full model including the covariates (Full.R2) = 0.012, Variance explained by the covariates only (Null.R2) = 0.003, PRS p-value of the model fit (p) = 0.001.

### Sample characteristics

The total sample comprised 1,068 participants, including 199 individuals at risk for BD and 869 healthy controls. At the time of the interview, participants (36.3% male, 63.7% female) ranged in age from 18 to 50 years, with an mean age of 28.32 years (SD = 7.67). As shown in Table 1, the groups did not differ significantly in sex distribution (χ²(1) = 1.63, *p* =.20), whereas the control group was significantly older than the at-risk group (*t*(551) = − 10.99, *p* <.001; Welch’s correction applied).

**Table 1:** Demographic data of ScreenBD at-risk (*N* = 199) as compared to controls (*N* = 869)

Demographics	ScreenBD at-risk (*N* = 199)	Control (*N* = 869)	Test	*p*
Sex (%)			χ² = 1.58	0.21
Female	59.8%	64.6%		
Male	40.2%	35.4%		
Age (years)	24.65 ± 4.34 SD	29.16 ± 8.011 SD	*t* (551)= −10.99	< 0.001

Control = healthy control, BD = Bipolar disorder, SD = standard deviation

### EPIbipolar and its subscales with genetic risk of BD

To capture all individuals with a potential risk for BD, participants classified as ‘low risk’, ‘high risk’ and ‘ultra high risk’ according to EPIbipolar were combined into a single ‘EPI*bipolar* at risk for BD’ category. BD-PRS score was significantly associated with EPI*bipolar* at risk (OR = 1.35, 95% CI [1.14, 1.59] vs. controls. Binary logistic regression for the EPI*bipolar* subscales showed BD-PRS was also associated with positive family history (FH) for BD vs. control status (OR = 1.62, 95% CI [1.07, 2.46], specific sleep and circadian rhythm disorders vs. control status (OR = 1.28, 95% CI [1.05, 2.57], cyclothymia with constant activation vs. control status (OR = 2.26, 95% CI [1.26, 3.66] depressive characteristics vs. control status (OR = 1.32, 95% CI [1.11, 1.58], positive FH for schizophrenia, schizoaffective disorder or MDD (OR = 1.34, 95% CI [1.08, 1.66], MDD vs. control status (OR = 1.36, 95% CI [1.14, 1.61], functional impairment vs. control status (OR = 1.27, 95% CI [1.07, 1.51], and episodic course vs. control status (OR = 1.34, 95% CI [1.10, 1.631]. The association of BD-PRS with cyclothymia with increasing activation vs. control group, hypomanic syndrome vs. control group, ADHD vs. control group and substance misuse were not significant. None of the analyzed (significant) risk factors showed an association with any of the first five principal components (PC1-PC5). For a summary of the regression coefficients, see Table 2.

**Table 2:** Associations of the BD-PRS with EPI*bipolar* risk factors

EPIbipolar Risk Factors	ß	SE	*P*	OR	95% CI	Nagelkerke’s *R*² observed
At-risk for BD (*N* = 1048, *n* = 177)						
BD-PRS	0.30	09	< 0.001	1.35	1.14–1.59	0.03
FH BD (*N* = 896, *n* = 25)						
BD-PRS	0.48	0.21	0.023	1.62	1.07–2.46	0.04
Increasing Cyclothmyia (*N* = 912, *n* = 41)						
BD-PRS	0.15	0.16	0.362	1.16	0.84–1.60	0.01
Hypomanic Syndrome (*N* = 915, *n* = 43)						
BD-PRS	0.09	0.16	0.560	1.10	0.80–1.50	0.02
Specific sleep and circadian rhythm disorders (N = 980, *n* = 109)						
BD-PRS	0.25	0.10	0.016	1.28	1.05–1.57	0.02
Consistent cyclothymia (*N* = 886, *n* = 15)						
BD-PRS	0.77	0.27	0.005	2.15	1.26–3.66	0.07
Depressive characteristics (*N* = 1026, *n* = 155)						
BD-PRS	0.28	0.90	0.002	1.32	1.11–1.58	0.03
FH Schizophrenia or MDD (*N* = 967, *n* = 96)						
BD-PRS	0.29	0.11	0.009	1.34	1.08–1.66	0.02
MDD (*N* = 1037, *n* = 168)						
BD-PRS	0.31	0.088	< 0.001	1.36	1.14–1.61	0.03
ADHD (*N* = 938, *n* = 67)						
BD-PRS	0.11	0.13	0.384	1.12	0.87–1.44	0.01
Functioning impairment (*N* = 1033, *n* = 162)						
BD-PRS	0.24	0.09	0.006	1.27	1.07–1.53	0.02
Episodic course (*N* = 988, *n* = 117)						
BD-PRS	0.29	0.10	0.004	1.34	1.10–1.63	0.02
Substance misuse (*N* = 885, *n* = 14)						
BD-PRS	0.06	0.27	0.820	1.06	0.63–1.80	0.01

Binary logistic regressions were adjusted ancestry PCs 1–5. BD = Bipolar Disorder, FH = Family History, ADHD = attention deficit hyperactivity disorder, MDD = major depressive disorder, CI = confidence interval, PC = principal component. N = number of participants; n = number of participants meeting the criteria

### BPSS-FP and BARS criteria with genetic risk of BD

The BD-PRS was associated with any risk group in BARS criteria (OR = 1.26, 95% CI [1.05, 1.52]) vs. no risk group, but not with any BPSS-FP syndrome.

**Table 3:** Associations of the BD-PRS with BPSS-FP syndrome

BPSS-FP any prodrom (*N* = 879, *n* = 65)	ß	SE	*P*	OR	95% CI	Nagelkerke’s *R*² observed
BD-PRS	− 0.30	0.79	0.70	0.74	0.16–3.48	0.29

Binary logistic regressions were adjusted ancestry PCs 1–5. CI = confidence interval, PC = principal component. N = number of participants; n = number of participants meeting the criteria

**Table 4:** Associations of the BD-PRS with BARS criteria

BARS criteria no vs. any risk group (*N* = 1006,*n* = 137)	ß	SE	*P*	OR	95% CI	Nagelkerke’s *R*² observed
BD-PRS	0.23	0.09	0.01	1.26	1.05–1.52	0.018

Binary logistic regressions were adjusted ancestry PCs 1–5. BAR: Bipolar At-Risk, BARS: extended BAR, CI = confidence interval. N = number of participants; n = number of participants meeting the criteria

## Discussion

Our results suggest that genetic risk, as captured by PRS, and clinical risk indicators identified through early detection tools partly converge. Rather than acting independently, they appear to reflect overlapping aspects of vulnerability. This overlap may explain why instruments such as EPI*bipolar* and BARS are sensitive to genetic loading in specific symptom domains.

When analyzed on their own, the BD-PRS were significantly associated with the key risk-measures in our at-risk sample, indicating they index a portion of the underlying liability. Although we did not test a full combined risk-prediction model, previous work suggests that genetic and clinical risk indicators are only partially overlapping (Mistry et al. 2018). Thus, the findings support the view that PRS might provide complementary rather than redundant information to clinical markers. Future work should test directly whether adding PRS to clinical predictors improves prediction of emerging illness or conversion in at-risk populations.

*PRS and* EPI*bipolar*.

The significant associations observed between BD-PRS and EPI*bipolar* — including positive family history (FH) for BD, sleep and circadian rhythm disturbances, depressive characteristics, functional impairment, and episodic course — suggest that genetic predisposition can be meaningfully linked to prodromal symptoms (see Supplementaty Table S2). The significant associations between BD-PRS and EPI*bipolar*, including a positive family history (FH) for BD, sleep and circadian rhythm disturbances, depressive features, functional impairment, and an episodic course, suggest that a genetic predisposition can plausibly be associated with prodromal symptoms (see Supplementaty Table S2). Instruments like EPI*bipolar*, which explicitly incorporate FH as a key predictor, exemplify how such integration can stratify risk more effectively.

Nevertheless, certain phenotypic indicators, including ADHD, hypomanic syndrome, increasing cyclothymia and substance abuse, did not demonstrate a significant association with BD-PRS. Our data did not reproduce the genetic overlap between ADHD and BD that has been described in previous studies (Biere et al. 2020). This discrepancy may be partly explained by the limited ability of the BD-PRS to detect genetic influences other than those specifically related to BD, as well as the limited statistical power for detecting associations in subgroups that are less prevalent in the sample. These results point to the need for more research into how distinct genetic and environmental factors shape specific phenotypes.

### PRS and BPSS-FP vs. PRS and BARS criteria

The association between BD-PRS and prodromal syndromes varies depending on the criteria and tools employed for assessment. Within BPSS-FP, no significant relationship was observed between BD-PRS and the Attenuated Mania Symptom Syndrome (AMSS), which focuses on subthreshold manic symptoms. This finding is in line with the results from EPIbipolar, where similar subthreshold manic symptoms showed no significant association with BD-PRS. The results suggest that these symptoms may not strongly correlate with the genetic predispositions captured by BD-PRS and may potentially limit their utility for predicting BD risk.

In contrast, the “Genetic Mania Risk and Deterioration Syndrome (GMRDS)” within BPSS-FP also showed no significant association with BD-PRS (Supplementaty Table S3). This is remarkable since other early detection instruments, such as EPIbipolar and BARS, identified significant associations between familial history (FH) and BD-PRS. The absence of such a relationship in GMRDS may reflect its small sample size (*n* = 2), which severely limits statistical power, rather than an inherent lack of association. While theoretical links between genetic risk and GMRDS exist, these findings underscore the necessity of larger and more representative samples for improved evaluation of the association between genetic predisposition and phenotypic indicators within this specific syndrome.

Broader assessment tools, such as the BARS criteria, showed a stronger association with BD-PRS. BARS integrates a range of factors, including subthreshold manic symptoms, depressive characteristics, and genetic predispositions, that identify BD-PRS as a significant predictor of overall risk group classification (OR = 1.26, 95% CI [1.05, 1.52]) (Supplementaty Table S4). BARS appears to have greater sensitivity to genetic risk compared to the BPSS-FP criteria, which target specific prodromal syndromes like AMSS. At the same time, BPSS-FP might still capture associations with syndromes such as GMRDS, but larger and more balanced samples will be needed to examine this in detail.

### Limitations and future directions

There are several limitations that must be considered. At first, the sample size in this study was relatively small, which may have limited the statistical power to detect weaker associations. A larger cohort would be needed to validate the findings and enhance the robustness of the results. Furthermore, the comparison group in this analysis was conservatively chosen to ensure robust and reliable results, following the recommendations of Kendler et al. (2020). The interpretability of findings may be limited since no genomic biomarkers were collected for the control group in the Early BipoLife study. Another limitation is that the at-risk and control samples were genotyped in two independent studies using different versions of the Illumina Global Screening Array (GSA MD v1 and v3). The datasets were processed separately, merged after initial quality control, and then underwent the same QC and imputation procedures. While this approach minimizes platform-related differences, small batch effects cannot be entirely ruled out. Future studies would benefit from integrating genetic data a priori into their study designs to address these gaps and to strengthen analytic precision.

Although indicators such as ADHD and hypomanic syndrome did not show significant associations, we observed meaningful relationships between BD-PRS and several EPI*bipolar* subscales. This highlights the potential specificity of BD-PRS for certain phenotypic markers and calls for further examination in larger cohorts. This may reflect a genuine specificity of BD-PRS for certain risk markers, or alternatively a lack of statistical power to detect small effects across all phenotypes. The absence of associations between BD-PRS and BPSS-FP syndromes further raises questions about the ability of mood-related prodromal symptoms to indicate BD risk. As noted by Hauser and Correll (2013) and Martini et al. (2024), mood fluctuations such as depressive or hypomanic episodes are common across mental disorders and even in the general population, which limits their specificity compared with subthreshold psychotic symptoms. This stresses the importance of combining multiple risk factors, including genetic predispositions and functional impairments, to improve early detection strategies. Future research could examine which clinical features interact most strongly with PRS to improve the precision of BD risk prediction and the specificity of early detection tools.

### Clinical implications

The results from this study suggest that integrating genetic data with early detection tools may improve our understanding of risk factors for BD. However, caution is still recommended when considering the use of PRS for clinical purposes. At this stage, PRS should not be viewed as a standalone diagnostic tool. Instead, it should be seen as a complementary measure that may provide additional context to phenotypic assessments. The ethical considerations surrounding genetic testing, such as privacy concerns, stigma, and the implications of false positive results, also need to be addressed. Clinicians should be trained to carefully interpret genetic data and integrate it alongside traditional diagnostic tools, in order to ensure that decisions about early intervention and care are holistic and evidence-based.

## Conclusion

This study contributes to the growing body of evidence which supports the integration of genetic and phenotypic data to detect BD at an early stage. The significant associations found between BD-PRS and specific risk factors suggest that PRS may serve as an adjunct to conventional diagnostic tools to improve pre-diagnostic risk stratification. However, further research is needed to refine these approaches, particularly with regard to phenotypic markers that are most predictive in combination with PRS. While these results are encouraging, their practical utility for early diagnosis and personalized treatment of BD remains to be fully validated.

## Supplementary Information

Supplementary Material 1

## Data Availability

The datasets presented in this article are not readily available because participants of the study did not give permission to publish their genome-wide data, based on privacy regulations. Requests to access the datasets should be directed to Reif@med.uni-frankfurt.de.
