# Supplementary material for: Advancing the prediction of factors associated with bipolar disorder risk: utilizing early recognition tools and polygenic risk scores
Source: Int J Bipolar Disord. 2025 Dec 10;14:2. doi: 10.1186/s40345-025-00404-8 (PMC12804565; doi:10.1186/s40345-025-00404-8)
Supplement: Supplementary file 1 — Supplementary Material 1 [file 40345_2025_404_MOESM1_ESM.docx]

**Anhang:**

Table 2 | Associations of the BD-PRS with EPI*bipolar* risk factors

|  | Risk Factor | *ß* | *SE* | *P* | *OR* | 95% CI | Nagelkerke’s *R*² observed |
| --- | --- | --- | --- | --- | --- | --- | --- |
|  | EPI*bipolar*  low + high risk  *(N = 1048, n = 177)* | |  |  |  |  | .03 |
|  | BD-PSS | .30 | .09 | < .001* | 1.35 | 1.14–1.59 |  |
|  | PC 1 | -3.67 | 2.42 | .13 | .03 | .00–2.95 |  |
|  | PC 2 | 3.89 | 2.56 | .13 | 49.01 | .33–7348.27 |  |
|  | PC 3 | 3.54 | 2.71 | .19 | 34.49 | .17–7040.04 |  |
|  | PC 4 | .73 | 2.74 | .79 | 2.07 | .01–439.86 |  |
|  | PC 5 | − .16 | 2.66 | .95 | .85 | .01–157.87 |  |
|  | Family history BD *(N = 1068, n = 25)* |  |  |  |  |  |  |
|  | BD-PRS | .48 | .21 | .023 | 1.62 | 1.07–2.46 | .04 |
|  | PC 1 | -2.73 | 5.76 | .636 | .07 | .07–5258.94 |  |
|  | PC 2 | 3.93 | 5.97 | .511 | 50.69 | .001–6086174.80 |  |
|  | PC 3 | 4.97 | 6.86 | .469 | 144.06 | .001–100056852.48 |  |
|  | PC 4 | 9.43 | 6.52 | .148 | 12458.15 | .000–4424005269.8 |  |
|  | PC 5 | .52 | 6.65 | .937 | 1.69 | .001–765572.93 |  |
|  | Increasing Cyclothmyia *(N = 1068, n = 41)* |  |  |  |  |  |  |
|  | BD-PRS | .15 | .16 | .36 | 1.16 | .84–1.60 | .01 |
|  | PC 1 | -4.76 | 4.34 | .27 | 0.01 | .000–42.15 |  |
|  | PC 2 | − .40 | 5.37 | .94 | .67 | .000–24892.15 |  |
|  | PC 3 | 1.61 | 5.40 | .78 | 4.99 | .000–195421.11 |  |
|  | PC 4 | -4.69 | 5.32 | .38 | .01 | .000–310.17 |  |
|  | PC 5 | -4.24 | 5.25 | .42 | .01 | .000–425.26 |  |
|  | hypomanic syndrome  *(N = 1068, n = 43)* |  |  |  |  |  |  |
|  | BD-PRS | .09 | .16 | .56 | 1.10 | .80–1.50 | .02 |
|  | PC 1 | -5.20 | 4.01 | .19 | .01 | .001–14.17 |  |
|  | PC 2 | -2.48 | 4.71 | .60 | .08 | .001– 858.95 |  |
|  | PC 3 | -1.73 | 5.06 | .73 | .18 | .000–3615.14 |  |
|  | PC 4 | 5.58 | 4.95 | .26 | 263.74 | .016–4337496.57 |  |
|  | PC 5 | 4.77 | 5.20 | .36 | 117.68 | .004–3164063.16 |  |
| specific sleep and circadian rhythm disorders *(N = 1068, n = 109)* | | |  |  |  |  |  |
| BD-PRS | | .25 | .10 | .016 | 1.28 | 1.05–1.57 | .02 |
| PC 1 | | -4.61 | 2.83 | .104 | .010 | 1.047–1.572 |  |
| PC 2 | | 2.67 | 3.17 | .399 | 14.387 | .000–2.560 |  |
| PC 3 | | 2.49 | 3.34 | .457 | 11.997 | .029–7105.884 |  |
| PC 4 | | -1.49 | 3.36 | .658 | .227 | .017–163.529 |  |
| PC 5 | | .77 | 3.24 | .811 | 2.169 | .04–1240.5433 |  |
| Consistent cyclothymia  *(N = 1068, n = 15)* | |  |  |  |  |  |  |
| BD-PRS | | .77 | .27 | .005 | 2.15 | 1.263–3.657 | .07 |
| PC 1 | | 4.01 | 11.05 | .717 | 55.32 | .000–1.419E + 11 |  |
| PC 2 | | .05 | 9.44 | .996 | 1.05 | .001–114108819.15 |  |
| PC 3 | | -5.23 | 8.85 | .555 | .01 | .001–181667.36 |  |
| PC 4 | | -6.61 | 8.75 | .450 | .00 | .000–37695.04 |  |
| PC 5 | | -1.53 | 8.41 | .855 | .22 | .000–3118031.54 |  |
| Depressive characteristics  *(N = 1068, n = 155)* | |  |  |  |  |  |  |
| BD-PRS | | .28 | .90 | .002 | 1.32 | 1.11–1.58 | .03 |
| PC 1 | | -4.29 | 2.48 | .083 | .014 | .00–1.76 |  |
| PC 2 | | 3.61 | 2.68 | .178 | 36.94 | .19–7058.85 |  |
| PC 3 | | 3.07 | 2.84 | .280 | 21.47 | .08–5632.09 |  |
| PC 4 | | .26 | 2.90 | .929 | 1.29 | .00–380.16 |  |
| PC 5 | | .14 | 2.82 | .961 | 1.15 | .01–285.66 |  |
| Family history of Schizo. or MDD  *(N = 1068, n = 96)* | |  |  |  |  |  |  |
| BD-PRS | | .29 | .11 | .009 | 1.34 | 1.075–1.656 | .02 |
| PC 1 | | -2.31 | 3.19 | .470 | .10 | .000–52.011 |  |
| PC 2 | | 2.70 | 3.31 | .413 | 14.93 | .02–9710.18 |  |
| PC 3 | | 3.20 | 3.52 | .363 | 24.54 | .03–24169.34 |  |
| PC 4 | | 4.14 | 3.48 | .235 | 62.79 | .07–57949.59 |  |
| PC 5 | | 2.30 | 3.45 | .505 | 9.99 | .01–8673.69 |  |
| MDD  *(N = 1037, n = 168)* | |  |  |  |  |  |  |
| BD-PRS | | .31 | .09 | < .001 | 1.36 | 1.14–1.61 | .03 |
| PC 1 | | -4.20 | 2.40 | .080 | .015 | .00–1.66 |  |
| PC 2 | | 3.49 | 2.61 | .181 | 32.83 | .20–5489.91 |  |
| PC 3 | | 2.67 | 2.76 | .333 | 14.46 | .07–3237.54 |  |
| PC 4 | | .37 | 2.80 | .896 | 1.44 | .01–345.23 |  |
| PC 5 | | − .04 | 2.73 | .988 | .96 | .01–201.63 |  |
| ADHD  *(N = 1068, n = 67)* | |  |  |  |  |  |  |
| BD-PRS | | .11 | .13 | .38 | 1.12 | .87–1.44 | .01 |
| PC 1 | | -4.74 | 3.40 | .16 | .01 | .00–6.85 |  |
| PC 2 | | 2.35 | 3.94 | .55 | 10.50 | .01–23471.32 |  |
| PC 3 | | 3.36 | 4.27 | .43 | 28.83 | .01–124642.63 |  |
| PC 4 | | .47 | 4.20 | .91 | 1.60 | .00–5945.74 |  |
| PC 5 | | -2.65 | 4.18 | .53 | .07 | .00–254.50 |  |
| Functioning impairment  *(N = 1068, n = 162)* | |  |  |  |  |  |  |
| BD-PRS | | .24 | .09 | .006 | 1.27 | 1.07–1.51 | .02 |
| PC 1 | | -3.84 | 2.47 | .119 | .021 | .00–2.71 |  |
| PC 2 | | 2.70 | 2.66 | .310 | 14.81 | .08–2695.51 |  |
| PC 3 | | 2.06 | 2.79 | .459 | 7.86 | .03–1844.90 |  |
| PC 4 | | .47 | 2.83 | .867 | 1.60 | .01–407.18 |  |
| PC 5 | | .81 | 2.75 | .770 | 2.24 | .01–493.70 |  |
| Episodic course  *(N = 988, n = 117)* | |  |  |  |  |  |  |
| BD-PRS | | .29 | .10 | .004 | 1.34 | 1.10–1.63 | .02 |
| PC 1 | | -4.63 | 2.71 | .088 | .01 | .00–1.99 |  |
| PC 2 | | 1.94 | 2.99 | .516 | 6.99 | .02–2455.89 |  |
| PC 3 | | -3.21 | 3.25 | .921 | .73 | .00–426.83 |  |
| PC 4 | | .91 | 3.21 | .777 | 2.48 | .01–1341.73 |  |
| PC 5 | | .54 | 3.22 | .687 | 1.72 | .00–947.08 |  |
| Substance misuse  *(N = 885, n = 14)* | |  |  |  |  |  |  |
| BD-PRS | | .06 | .27 | .820 | 1.06 | .63–1.80 | .01 |
| PC 1 | | 3.08 | 9.63 | .749 | 21.79 | .00–3404876307.1 |  |
| PC 2 | | 6.71 | 8.04 | .404 | 822.69 | .00–5737661415.2 |  |
| PC 3 | | -7.24 | 9.43 | .442 | .00 | .00–75856.28 |  |
| PC 4 | | 3.75 | 8.81 | .671 | 42.47 | .00–1343836877.0 |  |
| PC 5 | | -2.90 | 9.75 | .766 | .06 | .00–11021474.53 |  |

*Note.* Binary logistic regressions were adjusted ancestry PCs 1–5. BD = Bipolar Disorder, FH = Family History, ADHD = attention deficit hyperactivity disorder, MDD = major depressive disorder, CI = confidence interval, PC = principal component. N = number of participants; n = number of participants meeting the criteria.

Table 3 | Associations of the BD-PRS with BPSS-FP syndrome

| Risk Factor | *ß* | *SE* | *P* | *OR* | 95% CI | Nagelkerke’s *R*² observed |
| --- | --- | --- | --- | --- | --- | --- |
| BPSS any prodrom *(N = 939, n = 63)* |  |  |  |  |  |  |
| BD-PRS | − .301 | .79 | .70 | .74 | .16–3.48 | .287 |
| PC 1 | -202.79 | 79.62 | .01 | .00 | .00–.00 |  |
| PC 2 | 20.55 | 31.98 | .52 | 839435282.96 | .00–1.3882250545480788e + 36 |  |
| PC 3 | -6.09 | 27.23 | .82 | .00 | .00–3.415754697e + 20 |  |
| PC 4 | 30.64 | 28.85 | .29 | 2.03167164354e + 13 | .00–7.337793564e + 37 |  |
| PC 5 | -165 | 23.65 | .95 | .19 | .00–2.621196476e + 19 |  |

*Note.* Binary logistic regressions were adjusted ancestry PCs 1–5. CI = confidence interval, PC = principal component. N = number of participants; n = number of participants meeting the criteria.

Table 4 | Associations of the BD-PRS with BAR criteria

| Risk Factor | *ß* | *SE* | *P* | *OR* | 95% CI | Nagelkerke’s *R*² observed |
| --- | --- | --- | --- | --- | --- | --- |
| BARS criteria no vs. any risk group *(N = 869, n = 137)* |  |  |  |  |  |  |
| BD-PRS | .233 | .094 | .013 | 1.262 | 1.050–1.517 | .018 |
| PC 1 | -3.925 | 2.624 | .135 | .020 | .000–3.381 |  |
| PC 2 | 1.996 | 2.895 | .490 | 7.359 | .025–2141.492 |  |
| PC 3 | 3.265 | 3.029 | .281 | 26.168 | .069–9913.620 |  |
| PC 4 | − .142 | 3.038 | .963 | .868 | .002–334.180 |  |
| PC 5 | .399 | 2.975 | .893 | 1.490 | .004–507.401 |  |

*Note.* Binary logistic regressions were adjusted ancestry PCs 1–5. BAR: Bipolar At-Risk, BARS: extended BAR, CI = confidence interval. N = number of participants; n = number of participants meeting the criteria.
